# On the Relationship between MRI and Local Field Potential Measurements of Spatial and Temporal Variations in Functional Connectivity

**DOI:** 10.1038/s41598-019-45404-8

**Published:** 2019-06-20

**Authors:** Zhaoyue Shi, Don M. Wilkes, Pai-Feng Yang, Feng Wang, Ruiqi Wu, Tung-Lin Wu, Li Min Chen, John C. Gore

**Affiliations:** 1 Department of Biomedical Engineering, Vanderbilt University, Nashville, TN 37235 USA; 2 Vanderbilt University Institute of Imaging Science, Nashville, TN 37232 USA; 3 Department of Electrical Engineering and Computer Science, Vanderbilt University, Nashville, TN 37235 USA; 4 Department of Radiology and Radiological Sciences, Vanderbilt University, Nashville, TN 37232 USA; 5 Department of Physics and Astronomy, Vanderbilt University, Nashville, TN 37235 USA

**Keywords:** Computational biophysics, Cortex

## Abstract

Correlations between fluctuations in resting state BOLD fMRI signals are interpreted as measures of functional connectivity (FC), but the neural basis of their origins and their relationships to specific features of underlying electrophysiologic activity, have not been fully established. In particular, the dependence of FC metrics on different frequency bands of local field potentials (LFPs), and the relationship of dynamic changes in BOLD FC to underlying temporal variations of LFP correlations, are not known. We compared the spatial profiles of resting state coherences of different frequency bands of LFP signals, with high resolution resting state BOLD FC measurements. We also compared the probability distributions of temporal variations of connectivity in both modalities using a Markov chain model-based approach. We analyzed data obtained from the primary somatosensory (S1) cortex of monkeys. We found that in areas 3b and 1 of S1 cortex, low frequency LFP signal fluctuations were the main contributions to resting state LFP coherence. Additionally, the dynamic changes of BOLD FC behaved most similarly to the LFP low frequency signal coherence. These results indicate that, within the S1 cortex meso-scale circuit studied, resting state FC measures from BOLD fMRI mainly reflect contributions from low frequency LFP signals and their dynamic changes.

## Introduction

Functional MRI based on detecting BOLD (blood oxygenation level-dependent) signal changes is a well-established neuroimaging technique for detecting and spatially characterizing brain regions that change their levels of activity in response to specific external tasks^1–3^. In addition, the identification of patterns of correlated, low frequency fluctuations in BOLD signals in a resting state has provided a remarkable tool to assess functional connectivity between regions in the brain, providing insights into how distributed cortical areas work together to achieve specific functions^4–7^. However, BOLD signals are produced by hemodynamic effects that are only indirect indicators of underlying neural activity. Previous studies of the relationships between simultaneously recorded electrophysiological activity and hemodynamic changes in sensory systems have suggested that BOLD signals temporally correlate strongly with local field potentials^8–11^. Furthermore, our recent studies have indicated that at a columnar level, BOLD signals recorded at high magnetic field faithfully reflect underlying neuronal activity during stimulation, and the spatial profiles of local correlations in BOLD signals match those of LFP signals from the same region^12^.

The interpretation and application of fMRI-based functional connectivity studies rely on our understanding of the precise relationships between synchrony of local neural activity and correlated fluctuations in BOLD signals. To date, few studies have directly investigated the local spatial and temporal dynamic relationships between resting state BOLD signals and different frequency bands of local field potentials^13^. While our own and other studies have revealed how resting state fMRI connectivity spatially corresponds to correlations between LFPs, there have been few previous reports of what specific characteristics of LFPs best match BOLD correlations, or what dynamic features in the frequency contents of LFPs best align with temporal changes in BOLD metrics. Previous non-human primate brain studies have found that different frequency bands of LFP activities are linked to different neural information processes^14^. Typically, prominent LFP oscillations span a broad range of frequencies that by convention is separated into specific delta (1–4 Hz), theta (5–8 Hz), alpha (9–14 Hz), beta (15–30 Hz), gamma low (30–50 Hz), gamma high (50–100 Hz), and gamma very high (100–150 Hz) bands. In stimulation or task conditions, there have been consistent reports of close relationships between increases in BOLD signal amplitudes and increases in power of gamma band LFP (30–150 Hz) of LFP signals^15,16^. In a resting state, however, the relationships between resting state BOLD and spontaneous LFP activity remain elusive. While some studies suggest that lower frequency band fluctuations (<20 Hz) may predominantly correspond to changes in BOLD signals^14,17,18^, others suggest that slow changes in the gamma band of LFP signals contribute most to the spontaneous fluctuations in BOLD signals^19,20^. Moreover, resting state correlations of BOLD signals between regions appear to vary over time, and it has been postulated that these apparent dynamic changes of fMRI connectivity represent another level of neural modulation^21–25^. To date, few studies have directly compared the dynamic variations of connectivity seen in fMRI with corresponding properties of LFPs, nor have they established which LFP frequency bands are most relevant to these BOLD temporal dynamic changes^26,27^. No study, to our knowledge, has compared directly both the spatial relationships and the dynamic features of functional connectivity between BOLD signals and different frequency bands of LFPs within a well-defined, mesoscale functional network in a resting state.

We previously reported comparisons of local spatial profiles at columnar level between stimulus-evoked amplitudes and resting state correlations for both BOLD signals and broadband LFPs^12^. Here we examine those relationships more closely and address two specific new questions. First, to what extent do temporal dynamic changes in functional connectivity in BOLD and LFPs show similar quantitative characteristics, which is important to interpret the significance of dynamic changes in resting state correlations. Second, what specific frequency bands of LFP coherences most parallel the dynamic features of BOLD functional connectivity in a resting state, which is currently unclear given previous studies implicating different origins and functional relevance of different LFP frequencies^14,17,18,28^.

Specifically, we acquired high resolution (sub-millimeter) BOLD images at high field (9.4T) and multielectrode array LFP recordings from the areas 3b and 1 of primary somatosensory cortex (S1) in non-human primates. We measured the spatial extents of BOLD activations in response to tactile stimuli as well as the spatial profiles of single-voxel local correlations in a resting state. We then quantitatively compared these with the spatial extents of LFPs in each of seven frequency bands, and their inter-electrode coherences, in the same conditions and brain regions. We also compared dynamic features of the resting state connectivity measures in both modalities by using a modified Markov chain model-based approach combined with a sliding window correlation analysis. We aimed to quantitatively compare the time-varying patterns of functional correlations between BOLD and frequency-specific LFPs in individual monkeys, and to determine what frequency ranges of the LFP coherence dominate changes in dynamic BOLD functional correlations in two S1 cortices.

## Results

### The spatial extents of frequency-specific LFPs in stimulation and resting states

**Figure 1:**
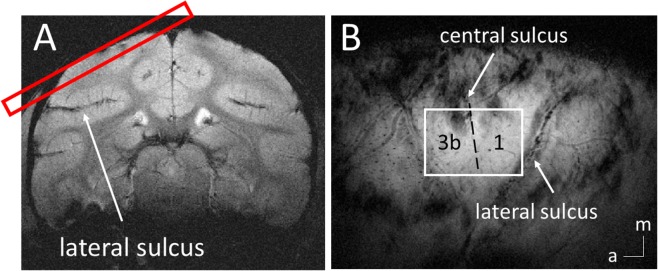
FMRI of S1 cortex of squirrel monkeys. (**A**) Top oblique imaging slice (red) is shown. (**B**) In an oblique coronal image acquired with T2* weighting, sulci and surface and transcortical vessels appear as dark lines and dots. a, anterior; m, medial.

**Figure 2:**
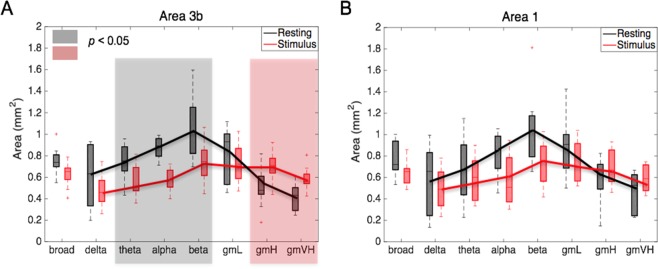
Comparison of spatial extents of LFP signals in eight frequency bands in areas 3b and 1, between resting state and stimulation. (**A**,**B**) The area values of areas 3b and 1, respectively, in both resting (black boxes) and stimulus (red boxes) conditions, significance at *p* < 0.05. (**A**) Wilcoxon signed-rank test, as shown in the grey and pink background). A total of 9 runs from three monkeys were analyzed for LFP measurements. Error bars indicate standard deviations.

Figure 1 shows the placement of a typical fMRI image slice and regions of interest (ROIs) in areas 3b and 1 in S1 cortex. Figure 2 shows the spatial variations in power and coherence characteristics of the LFP signals in different frequency bands during stimulation and resting states in two areas of S1 cortex. A Wilcoxon signed-rank test showed that in a resting state (black boxes), in area 3b, the local coherence profiles of LFP signals in the frequency ranges between 5 and 30 Hz were significantly broader in extent than those in the frequency ranges between 50 and 150 Hz. In addition, the local coherence profiles of the broadband (1–150 Hz) LFPs had no significant differences from those of low frequency (1–15 Hz) and gamma low (30–50 Hz) LFPs. Direct comparisons of the area values between stimulation and resting states showed that, in area 3b, the spatial extents of resting state electrode-electrode coherence in the frequency ranges between 5 to 30 Hz and the broad band were significantly wider than those of stimulation responses (Wilcoxon signed-rank test: *p* < 0.05; Fig. 2A, the grey background), while the spatial extents of resting state coherence in the frequency ranges between 50 and 150 Hz are significantly narrower than those of stimulation responses (*p* < 0.05; Fig. 2A, the pink background), a feature that was repeated but was not statistically significant in area 1 (Fig. 2B). These comparisons revealed that in S1, the local coherence profiles of broadband resting state LFPs were closer to those of low frequency LFP components than high frequencies. In addition, low frequency LFP signal fluctuations contributed most to the spatial extents of resting state LFP coherence, while high frequency LFP responses contributed most to the spatial extents of activation patterns.

**Figure 3:**
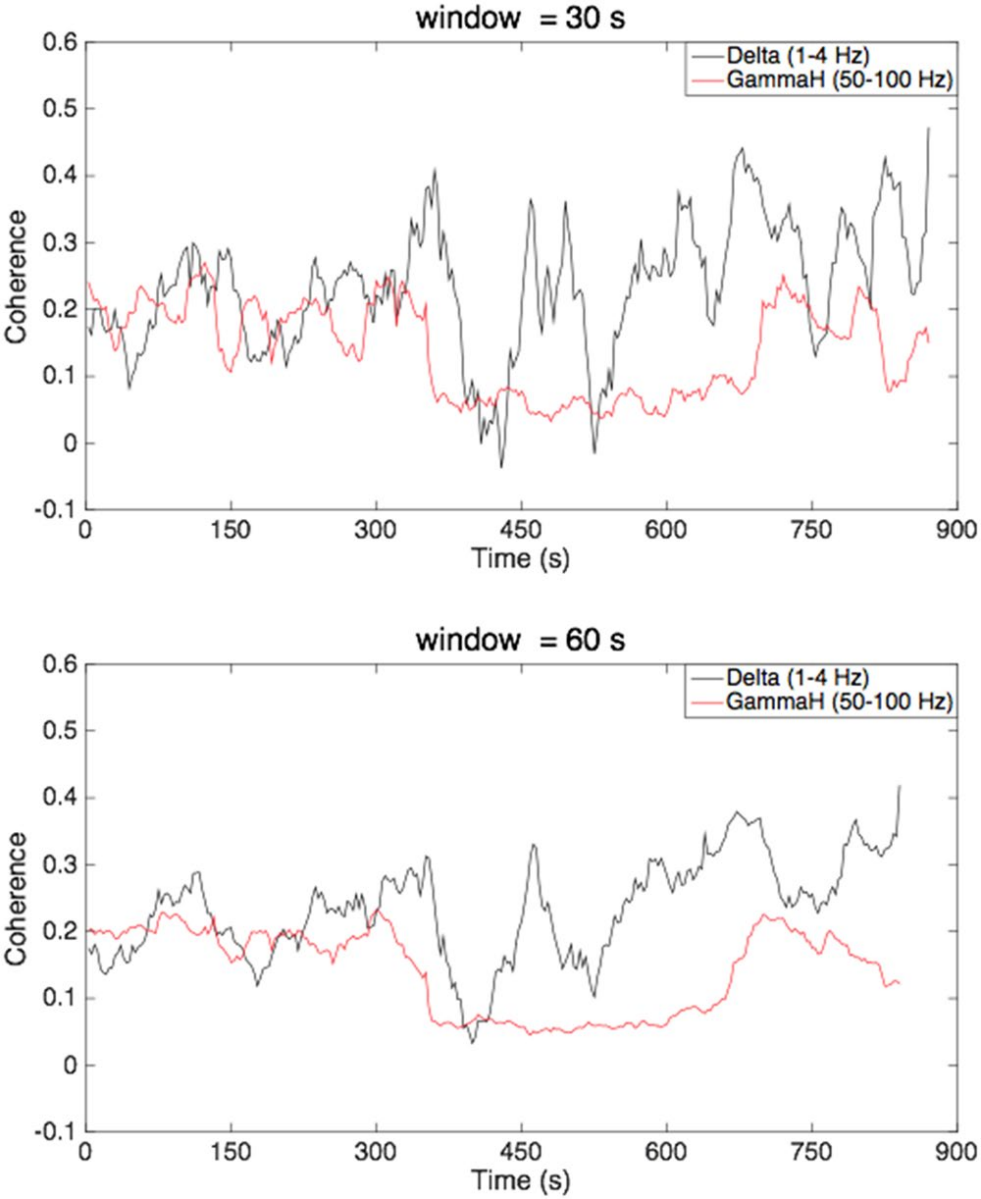
Examples of a sliding window coherence analysis between areas 3b and 1 from monkey (SM-TE), comparing delta band LFPs (black line: 1–4 Hz) and gamma high band LFPs (red line: 50–100 Hz), with a window size of 30 sec and 60 sec, respectively.

### The temporal dynamic features of inter-areal LFP coherence in different frequency bands

**Figure 4:**
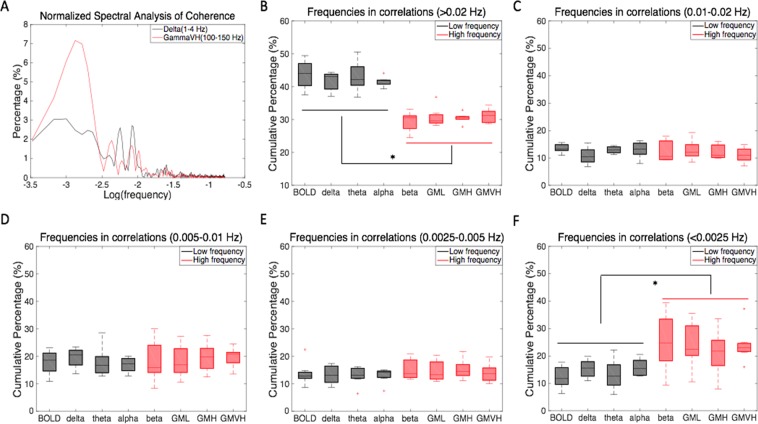
Group analysis on the Fourier spectral decomposition of the correlation between areas 3b and 1 in resting state BOLD signals and LFPs in seven frequency bands. (**A**) Normalized percentage of spectral analysis of sliding window coherence, comparing LFP delta (black) and gamma high (red) bands. (**B**–**F**) Cumulative percentage of variation in correlations from different frequency bands; (**B**) over 0.02 Hz; (**C**) between 0.01 and 0.02 Hz; (**D**) between 0.005 and 0.01 Hz; (**E**) between 0.0025 and 0.005 Hz; (**F**) less than 0.0025 Hz. *Wilcoxon signed-rank test at p < 0.05. A total of 9 runs from three monkeys were analyzed for BOLD and LFP measurements, respectively. Error bars indicate standard deviations.

Figure 3 shows an example of a sliding window coherence analysis between areas 3b and 1, comparing delta band LFPs (low frequency: 1-4 Hz) and gamma high band LFPs (high frequency: 50-100 Hz), with window sizes of 30 sec and 60 sec. The selection of a 60 sec window size was based on our previous studies^25^, but we also evaluated whether smaller window sizes would reveal additional apparent changes in resting state connectivity. The dynamic patterns of functional correlations were very similar. The low frequency LFP coherence changed more frequently than for high frequency components. We quantified this difference by analyzing the Fourier spectral composition of the coherence between areas 3b and 1 in resting state BOLD signals and frequency-specific LFPs. We compared the cumulative percentage of frequency components derived from resting state BOLD correlations with frequency-specific LFP coherences. Figure 4 shows a group analysis for LFPs in seven frequency bands. As shown in Fig. 4B, a Wilcoxon signed-rank test showed that variations in frequency components >0.02 Hz contributed significantly more to the resting state BOLD correlations and low frequency LFP coherence (1–15 Hz), than high frequency LFP coherence (15–150 Hz). From Fig. 4F, it can be seen that variations in frequency components between 0 and 0.0025 Hz made significantly greater contributions to the resting state high frequency LFP coherence than low frequency LFP coherence and BOLD correlations.

### Comparisons of resting state dynamic connectivity between BOLD and frequency-specific LFPs

**Figure 5:**
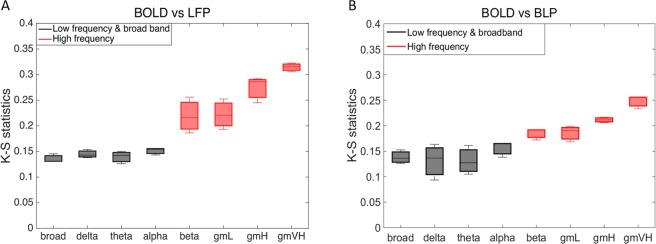
Group analysis on Kolmogorov-Smirnov statistic between BOLD fMRI correlations and their corresponding monkey’s LFP coherence and band-limited power correlation in eight frequency bands, between areas 3b and 1. (**A**) Dynamic changes in sliding window correlations in BOLD data were distributed more closely to low frequency LFPs (black, 1–15 Hz) and broad band LFPs (black boxes, 1–150 Hz) coherence, while differed from LFP coherence at high frequency bands (red boxes, 15–150 Hz). (**B**) Dynamic changes of BOLD functional correlations behaved most similarly to the low frequency BLP correlations (black, 1–15 Hz) when compared to the high frequency BLP correlations (red boxes, 15–150 Hz). A total of 9 runs from three monkeys were analyzed for BOLD, LFP, and BLP measurements, respectively. Error bars indicate standard deviations.

We also investigated the dynamic changes of functional connectivity in BOLD and frequency-specific LFPs using a Markov chain model-based approach combined with a two sample K-S statistic. We quantitatively compared the time-varying distributions of correlations between areas 3b and 1, between BOLD and LFP signals in eight frequency bands. Figure 5A showed the K-S statistic for group differences of fMRI correlations and their corresponding monkey’s LFP coherence between areas 3b and 1, in eight frequency bands. The correlations derived from resting state BOLD signals were distributed more similarly to the LFP coherence in broad band (1–150 Hz) and low frequency bands (1–15 Hz, black boxes), than to the LFP coherence in high frequency bands (30–150 Hz, red boxes), with substantial differences appearing between 50 and 150 Hz. Specifically, K-S values of correlation coefficient distributions between areas 3b and 1 of the BOLD signals and LFPs in broad band and low frequency bands were significantly smaller than those of the same BOLD signals and LFPs in high frequency bands (Wilcoxon signed-rank test at *p* < 0.01). These results indicate that within S1 cortex, the dynamic changes of BOLD functional correlations behaved more closely to those of low frequency than high frequency LFP coherences. We also applied the band-limited power (BLP, Fig. 5B) correlation analysis which extracts the envelop of raw LFP signals in various frequency bands^29^, and found that the dynamic changes of BOLD functional correlations behaved most similarly to the low frequency BLP correlations when compared to the high frequency BLP correlations, consistent with our LFP coherence analysis.

## Discussion

The aim of this study was to investigate in what frequency ranges LFP coherences were most similar in spatial extent and showed similar dynamic changes as BOLD functional connectivity in a resting state. Our data were obtained from areas 3b and 1 of S1 in nonhuman primates by sampling at columnar resolution. Currently, there is no consensus as to whether low or high frequency LFP signals correspond most closely to the spontaneous fluctuations in fMRI signals^17,18,20,27,29–31^. We compared the areas of local correlation profiles of resting state BOLD signals with those of ‘gold standard’ electrophysiological LFP measurements, and investigated the temporal dynamic changes in functional connectivity in both modalities. We found that within S1 cortex, low frequency LFP signal fluctuations were the dominant contributors to the spatial extent of resting state LFP coherence. In addition, our analysis of temporal variations in correlations showed that coherences of low frequency LFPs changed significantly more frequently than those of high frequency LFPs, and that the changes of BOLD functional connectivity over time behaved more similarly to those of LFP low frequency rather than higher frequency coherences. These results suggest that in S1 cortex, resting state BOLD dynamic connectivity is reflective more of variations in low frequency LFP coherence than high frequency LFP coherence.

The local coherence profile of LFP signals within S1 cortex in a resting state directly measures the contributions from the intrinsic spatial distribution of the neural activity involved, and potentially reflect how neurons spatially work together at a columnar level. No previous study, to our knowledge, has reported the spatial relationships of LFP signals in different frequency ranges during stimulation or in a resting state. We found that in a resting state, the local correlation profiles of LFP signals from the frequency ranges between 50 and 150 Hz were significantly smaller than those of the frequency ranges between 5 and 30 Hz. This suggests that in a resting state, neurons generating high frequency LFP signals (50–150 Hz) may intrinsically communicate more focally within a finer-scale cortical microcircuit^32–34^, than those generating low frequency LFP signals (1–15 Hz). In addition, evidence has shown that the neural oscillations of frequency-specific LFP signals may be generated by various types of neurons in different pathways^35–37^. The primary somatosensory cortex is a cortical region receiving a convergence of anatomical connections from structures in the cortico-thalamic and cortico-cortical loops^38–40^. Low frequency oscillations can occur in a cortico-thalamic feedback loop. These slow oscillations are thought to be modulated by global neuronal inputs^28,37,41^. However, high frequency LFPs, such as gamma-waves, most likely arise locally within cortical microcircuits that include pyramidal cells and interneurons^37,42–44^, and therefore, present a more constrained local spatial correlation profile. Our results also showed that in a resting state, the local correlation profiles of broadband LFPs were not significantly different from those of low frequency LFPs (1–15 Hz) and gamma low band (30–50 Hz). Furthermore, we directly compared the spatial profiles between stimulation and resting state conditions in both areas 3b and 1, respectively. We found that in area 3b, the spatial extents of resting state electrode-electrode coherence in the broad band (1–150 Hz) and the frequency ranges between 5 and 30 Hz were significantly larger than those of stimulation responses. However, in the frequency ranges between 50 and 150 Hz, the spatial extents of resting state coherence were significantly smaller than those of stimulation responses, a feature that was not statistically significant in area 1. These comparisons show that in S1 cortex, low frequency LFP signal fluctuations spatially dominate resting state LFP coherence, while stimulus responses are dominated by high frequency LFP responses. These relationships could potentially vary in different brain regions. For example, in the hippocampus, strong theta oscillations (5–8 Hz) are the most prominent feature in LFP signals and are believed to be critical for hippocampal-cortical interactions^45,46^, while in visual cortex, fast oscillations in the gamma band between 30 and 90 Hz are dominant during resting states^37,47^.

We identified seed voxels and electrodes for calculating resting state correlations in BOLD and LFP measurements separately, according to the stimulus-evoked activation maps in each monkey. In Figs 3 and 4, the resting state sliding window LFP coherence between areas 3b and 1 shows that coherences of low frequency (1–15 Hz) LFP signals change significantly more frequently than those of high frequency (15–150 Hz) LFP signals. As shown in Fig. 5, the correlations derived from resting state BOLD signals between areas 3b and 1 were distributed more similarly to the LFP coherence in broad band (1–150 Hz) and low frequency bands (1–15 Hz), than in high frequency bands (30–150 Hz). These results indicate that in a resting state, the dynamic changes of BOLD functional correlations behave more closely to those of low frequency than high frequency LFP coherences. In addition, the band-limited power correlation analysis also found that the dynamic changes of BOLD functional correlations behaved more similarly to the low frequency BLP correlations than the high frequency BLP correlations, consistent with our LFP coherence analysis. One possible hypothesis is that in S1 cortex, the frequency ranges from 0.01 to 0.1 Hz of resting state BOLD fMRI signals are reflective of the cortico-thalamic feedback loop. Another possible hypothesis, based on what is reported in the literature, is that the observed BOLD connectivities originate mainly from superficial layers^48,49^, and some electrophysiological studies have shown that delta (1–4 Hz) and alpha (9–14 Hz) generators are strongly represented in superficial layers^50,51^. It will be valuable to investigate the laminar profile of LFP signals in different frequency ranges, and the spatial and temporal relationships between cortical layer-specific BOLD signals and their corresponding LFPs. Lastly, it is worth noting that in animal studies, in order to obtain reliable interpretations of functional connectivity changes, it is necessary to estimate the effects of anesthetics on the resting state fMRI signals. In our lab’s previous publication^52^, the authors observed universal dose-dependent suppressive effects of isoflurane anesthesia on cortical regions. Although it is difficult to form a standard anesthesia range across studies, for the purposes of this study, we maintained an appropriate isoflurane level (0.7–0.8%) for imaging of the S1 cortex as stable as possible during all functional scans.

## Methods

### Animal preparation

Three squirrel monkeys (*Saimiri bolivians*, SM-ZT, SM-TE, SM-FZ) were included in this study and each underwent functional MR imaging, micro-electrode electrophysiological mapping and multi-channel microelectrode array recording sessions. Animals were pre-anesthetized with ketamine hydrochloride (10 mg/kg)/atropine (0.05 mg/kg) and then anesthetized with 0.5–1.5% of isoflurane delivered in a 30:70 O_2_:N_2_O mixture, to maintain a stable physiological condition for both MRI scans and electrophysiological acquisitions. Although the actual level of isoflurane may vary during the scan and across animals, we typically maintained a light level of anesthesia at ≈ 0.7–0.8% isoflurane during all functional acquisitions and electrophysiological signal recordings. The anesthetized animals were intubated and artificially ventilated. After intubation, each animal was placed in a custom-designed MR cradle with its head secured using ear and head bars. Lactated Ringer’s solution was infused intravenously (2–3 ml/h/kg) to prevent dehydration during the course of the study. Blood oxygen saturation and heart rate (Nonin, Plymouth, MN), electrocardiogram, end-tidal CO_2_ (ET-CO_2_; 22–26 mmHg; Surgivet, Waukesha, WI), and respiration (SA Instruments, Stony Brook, NY) were monitored and maintained at stable levels. Body temperature was monitored (SA Instruments) and maintained between 37.5 and 38.5 °C via a combination of a circulating water blanket (Gaymar Industries, Orchard Park, NY). Animals were carefully monitored from the time of induction of anesthesia until full recovery. Analgesic (buprenorphine) is for 3 days post-surgery. Detailed procedures have been described in our previous publication^12,53–55^. All experimental procedures were in compliance with and approved by the Institutional Animal Care and Use Committee of Vanderbilt University, and followed the guidelines of the National Institute of Health Guide for the care and use of laboratory animals.

### Stimulus protocol

The anesthetized animals’ fingers were secured by gluing small pegs to the fingernails and fixing these pegs firmly in plasticine (a brand name of modeling clay), leaving the glabrous surfaces available for vibrotactile stimulation by a blunt plastic probe (2 mm diameter) connected to a piezoelectric vibrator (Noliac, Kvistgaard, Denmark). The vibrators were driven by Grass S48 square wave stimulators (Grass-Telefactor, West Warwick, RI) at a rate of 8 Hz. For fMRI data acquisitions, vertical indentations (0.34 mm displacement) of a probe at 8 Hz rate (with 20-ms pulse duration) were presented as blocks of 30 sec on and then 30 sec off. Seven blocks were typically presented within one imaging run. Multiple runs were typically acquired. The timing of the presentation of stimuli was externally controlled by the MR scanner and was synchronized to image acquisition. The same stimulus presentation paradigm was applied during later electrophysiological recordings. Each stimulation trial consisted of 20 repetitions of 30 sec on/off blocks. Typically, 10 trials were collected for each digit. Detailed protocol has been described in our previous publication^12,25^.

### FMRI data acquisition and preprocessing

MR imaging was performed with a 9.4 T 21 cm bore magnet and Varian/Agilent Inova spectrometer (Varian Inc., Palo Alto, CA), using a 3-cm surface transmit-receive coil secured over S1 cortex. Scout images obtained using a fast gradient-echo sequence were used to define a volume covering S1 cortex (including areas 3a, 3b, 1 and 2) in which static magnetic field homogeneity was optimized, and to plan four oblique slices for structural and functional imaging. T_2_^*^-weighted gradient-echo structural images [repetition time (TR) = 200 ms; echo time (TE) = 16 ms; four slices, FOV = 35 × 35 mm^2^, 512 × 512 matrix; spatial resolution = 0.068 × 0.068 × 2 mm^3^; number of excitations = 6] were acquired to identify venous structures on the cortical surface to help locate S1 cortex and as structural features for co-registration of fMRI images (see Fig. 1 for the placement of fMRI image slice). Within each imaging session, multiple runs (up to 6) of stimulus-evoked and resting state fMRI data were acquired with identical image acquisition sequences (gradient echo-planar sequence; TR = 750 ms; TE = 16 ms, number of excitations = 4), and slice placement. Functional images were obtained at an imaging resolution of 0.274 × 0.274 × 2 mm^3^. Each high resolution stimulation run contained 150 continuous image volumes. For resting state fMRI runs, 300 continuous image volumes were acquired. MR images were reconstructed on the MR console (Varian VnmrJ) and exported to Matlab (Mathworks) for analysis^12^.

**Figure 6:**
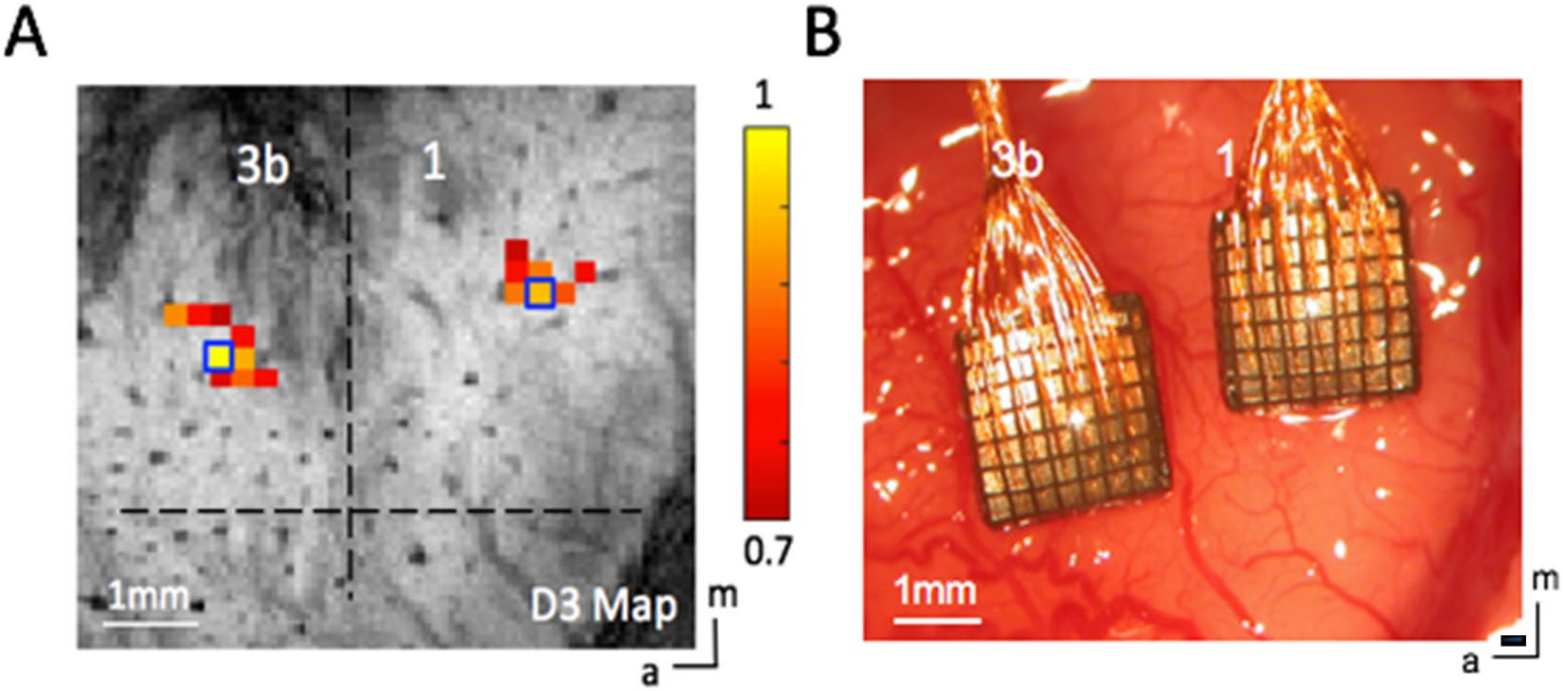
Stimulus-evoked fMRI activation map (**A**) Localization of activated digit regions in areas 3b and 1 of one representative squirrel monkey (SM-ZT) in response to vibrotactile stimulation of the D3 tip. Normalized percentage signal change maps were thresholded at 0.7. Dotted black lines indicate the approximate borders between areas 3b and 1, and between digit and face. Blue boxes show the selected seed voxels used in subsequent resting state functional connectivity analysis. (**B**) Optical image showing blood vessels and the two 7 × 7 multi-channel electrode arrays inserted in the corresponding digit regions of areas 3b and 1 under surgical microscope guidance in the same monkey (SM-ZT). (Scale bar, 1 mm.) a, anterior; m, medial.

Standard preprocessing steps were applied to the raw fMRI signals. Slice timing correction was performed after slice-by-slice motion correction. Six translation and rotation parameters were used to regress out temporal variations caused by motion. Time courses were drift corrected using a linear model fitted to each time course. The RETROICOR method was used to correct for physiological noise, using the respiration and cardiac patterns recorded during the scan^56^. No spatial smoothing was applied. Resting state BOLD signal time series were further filtered with a low-pass filter (0.01–0.1 Hz). The top image slice, in which areas 3b and 1 reside, was analyzed for activations. For stimulation runs, we computed activation maps based on the percentage of BOLD signal change between pre-stimuli periods (seven out of ten imaging volumes before stimulus onset) and stimuli presentation periods (10 image volumes). The activation response map (Fig. 6A) was then up-sampled to the anatomic MRI image resolution (0.068 × 0.068 mm^2^) for further sub-voxel processing and display. Within each fMRI stimulation run, the percentage of BOLD signal changes in each of the cortical areas (areas 3b and 1) was normalized to its own maximum value. Normalized percentage BOLD signal changes were then used in the group analyses. Detailed fMRI data acquisition and preprocessing procedures have been described in our previous publication^12^.

### Local field potentials recording and analysis

**Figure 7:**
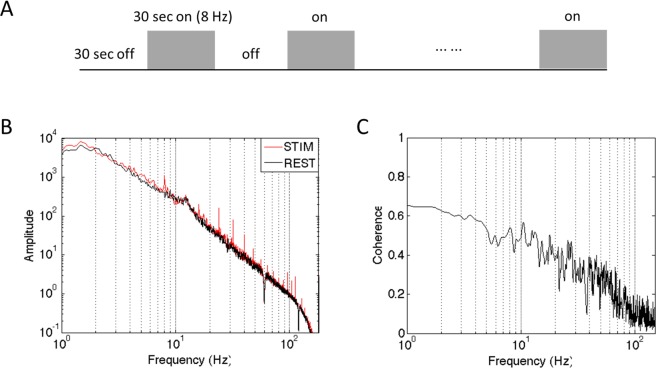
(**A**) The stimulation paradigm for both LFP and BOLD fMRI experiments. (**B**) Grand mean power spectrum of the raw LFP signal during an entire 8 Hz stimulation session, including stimulation trials and inter-trial rest, averaged over all 96 electrodes. (**C**) Pairwise cross coherence of the raw resting state LFP signals between area 3b and area 1 ROI seeds.

Electrophysiology data was recorded at the end of the study (no recovery). As seen in Fig. 6B, two 7 × 7 multichannel Utah electrode arrays with 98 channels total, electrode length of 1 mm, and electrode spacings of 400 μm were carefully implanted into the targeted area 3b and area 1 cortex using a pressure inserter. This process was guided by stimulus-evoked fMRI activation maps and blood vasculature patterns. Both spiking and LFP data were collected, and while spiking activity was present in some channels, we focused our analysis on LFP signals only in the present study. LFP signals were sampled at 500 Hz and later band-pass filtered into eight frequency ranges using a second order, zero-phase Chebyshev type-1 filter in Matlab. The frequency ranges were the following: delta (1–4 Hz), theta (5–8 Hz), alpha (9–14 Hz), beta (15–30 Hz), gamma low (30–50 Hz), gamma high (50–100 Hz), gamma very high (100–150 Hz) and broad band (1–150 Hz). For the stimulation condition (Fig. 7A), a time-frequency analysis was conducted to evaluate the temporal structure of the LFP signals and their stimulus response preferences. A Fourier transform was performed on the data from each trial and the resulting spectrograms were averaged (Fig. 7B). Computed spectrograms of stimulus-evoked LFPs were transformed into a dB scale (10 × log_10_) and used for further analysis. To estimate more accurately the mean power of the steady-state LFP responses, we excluded the data from the first 10 sec after the stimulus onset in our calculation, according to previous observations^57^. For LFP resting state coherence analysis, magnitude squared coherence in each frequency band was calculated between each selected seed electrode and all other electrodes (Fig. 7C). Detailed LFP recording and preprocessing procedures have been described in our previous publication^12^. Band-limited power was calculated as described thoroughly in the literature^29^.

### Measurements of the point spread functions of BOLD and LFP signals in stimulation and resting states

We quantified the spatial extents of activations and local correlations using the same approach as described in our previous publication^12^. To extend our previous analyses, the same fitting methods were used to quantify the spatial extents of LFP signals in each of the eight frequency bands. We identified activation foci in areas 3b and 1, whose shapes appear to be elliptical. In each animal, activation maps of three different digit tips (digits 2, 3, 4) were typically obtained. A threshold of 0.7 was used to identify the center of mass in each activation focus. The axis along the individual digit activation centers was then determined as the major (from lateral to medial) axis within each area (3b and 1). The perpendicular anterior-posterior direction of this axis was then determined as the minor axis. From the fitting, the major (in the lateral to medial direction) and minor (in the anterior to posterior direction) axes of the ellipse were determined, and all values above 0.2 of normalized percentage signal change along the major and minor axes within the sub-region were used in the subsequent Gaussian fitting. The point spread functions (PSFs, as indicated by the full-width of half maximum: FWHM) of the single digit tactile stimulation evoked BOLD fMRI activation maps were measured in both areas 3b and 1. The coordinates of the center of mass, and the major and minor axes of the ellipse were determined by fitting. The spatial distributions of percentage of BOLD signal changes along the major and minor axes were then fit with Gaussian functions. The FWHM of each fitted Gaussian was then computed. For resting state fMRI runs, ROI seeds were identified based on the vibrotactile stimulus-evoked activation maps. The voxels with the highest % BOLD signal change were chosen as the seeds for each digit in either areas 3b or 1 (Fig. 6A). Resting state BOLD signal time courses were extracted from the seed voxels and then used as the reference models in subsequent voxel-wise correlation analyses. Correlation coefficients were computed for each voxel surrounding the seed, and then local functional connectivity maps were generated for each seed. Identical spatial fitting procedures were applied to derive the FWHM and area of Gaussian PSFs of fitted local correlation profiles^12^.

To quantify the dimensions of LFP activities in each frequency band, the same ellipsoid Gaussian PSF (FWHM) fitting was performed^12^. Multi-channel electrode array response maps were computed based on the percentages of signal power changes between 20 sec of stimuli presentations and 20 sec of the pre-stimuli periods during single digit stimulations. Activation maps were then resampled at higher resolution (0.10 × 0.10 mm^2^) for further sub-voxel processing. Within each LFP stimulus recording run, the percentages of signal power changes of cortical activation areas were normalized to their maximum values during each run. For resting-state LFPs, seed electrodes were identified based on the array stimulus response maps as those showing the largest percentage signal power changes during single digit stimulations in both area 3b and area 1. Functional connectivity maps based on functional coherence between each seed and the rest of the electrodes were then computed. We then used the same fitting as described above to describe the spatial profiles of resting-state LFP coherences of this area.

### Quantification of temporal dynamics of resting state functional connectivity between areas 3b and 1 using a combined Markov chain model and sliding window approach

Previous studies have shown that resting state correlations in BOLD signals between regions may vary over time. Here we compared whether LFPs show similar dynamic changes, and examined which (if any) specific LFP frequencies most closely match those of fMRI signals. To do this, we consider the instantaneous degree of correlation between regions to represent a brain state, so that changes in connectivity correspond to transitions between brain states. The number of such states is unknown but we assume they may span the range between perfectly correlated and perfectly anti-correlated, and this range can then be divided into a finite number of discrete intervals. Measurements of the variations over time in correlation values allow the construction of a probability distribution of the states, along with probabilities for transitions from any one state to another. A discrete-time Markov chain model is a tool for representing probability distributions over a sequence of states in which the probability of moving to the next state depends only on the present and not on the previous states^58^.

**Figure 8:**
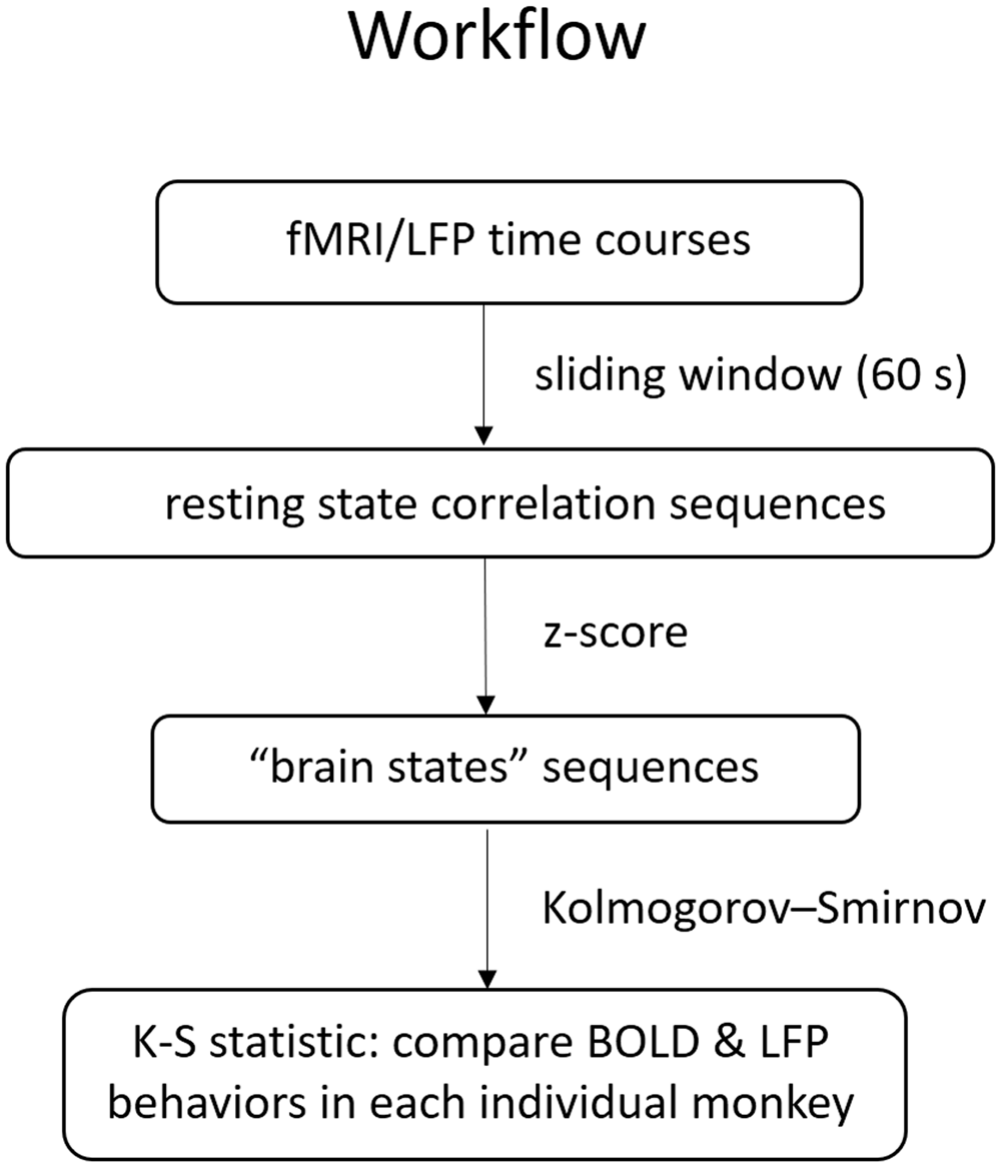
Workflow diagram of the Markov chain model-based approach.

Figure 8 shows a workflow diagram of the Markov chain model-based approach. A conventional sliding window technique provides a measure of dynamic changes in the correlation as estimated over the window length and is a method for capturing variations in inter-regional synchrony that has been used to investigate temporal dynamic changes in correlations between time courses in various modalities^22–25^. The sampling variability and statistical efficiency of the sliding window technique has been recently investigated^21,59^. In the current study, resting state correlations between the BOLD time series derived from areas 3b and 1 within S1 cortex were calculated using a sliding window with a window size of 60 sec. The selection of the window size was based on simulations and a two sample Kolmogorov-Smirnov (K-S) statistic in our previous studies^25^ which established this was sufficient to attenuate spurious variations while maintaining low frequency features of interest. The window was shifted in time in 3 sec increments (equal to time between image volumes) along the entire time series and the correlation coefficient was recalculated. As the sampling distribution of Pearson’s *r* is not normally distributed, correlation coefficients were converted to z-scores using Fisher’s z transform^25^. The z-scores from −1 to 1 were binned into 50 equal steps, which represent 50 putative brain states characterized by their differing degree of correlations between areas 3b and 1. The selection of 50 was arbitrary but shown to not be a critical parameter. A similar sliding window technique was used to classify the LFP signals. In this analysis, however, the magnitude squared coherence values for each frequency band were computed from the raw LFPs^29^ instead of Pearson’s correlation coefficients. We then quantified the resemblance of the temporal variations in the correlations between BOLD and frequency-specific LFP signals by constructing the empirical cumulative distribution functions (ECDFs) for jumps between states, which represent the probability functions for single-step transitions between different degrees of connectivity. A two-sample Kolmogorov-Smirnov (K-S) statistic^60^ was used to evaluate whether the BOLD and LFP ECDFs were derived from the same population with a specific distribution, based on measurements of the distance between two ECDFs. This test is sensitive to differences in both the location and shape of the ECDFs of the two samples^25,60^. Lower K-S values between BOLD and LFP in a certain frequency band suggest increased similarity in the nature of the dynamic changes in functional connectivity.

## Data Availability

The datasets generated during and/or analysed during the current study are available from the corresponding author on reasonable request.
